# Modulation of Antioxidant Compounds in Fruits of *Citrus reticulata* Blanco Using Postharvest LED Irradiation

**DOI:** 10.3390/biology12071029

**Published:** 2023-07-21

**Authors:** Giulia Costanzo, Ermenegilda Vitale, Maria Rosaria Iesce, Michele Spinelli, Carolina Fontanarosa, Roberta Paradiso, Angela Amoresano, Carmen Arena

**Affiliations:** 1 Department of Biology, University of Naples Federico II, Via Cinthia, 80126 Napoli, Italy; giulia.costanzo2@unina.it; 2 Department of Chemical Sciences, University of Naples Federico II, Via Cinthia, 80126 Napoli, Italy; 3 Department of Agricultural Sciences, University of Naples Federico II, Via Università 100, 80055 Portici, Italy; 4 NBFC-National Biodiversity Future Center, 90133 Palermo, Italy

**Keywords:** antioxidant capacity, carotenoids, flavonoids, light quality, light spectrum, Phlegrean mandarin, polyphenols

## Abstract

**Simple Summary:**

The study evaluated the effect of different light quality regimes applied during postharvest storage on the regulation of phenolic compounds, carotenoids, and antioxidant activity in the peel and pulp of Phlegrean mandarin fruits. After seven days of red–blue (RB) light regime exposure, the increase of antioxidants in both fruit components confirmed the effectiveness of the light quality modulation in enhancing plant phytochemical content. The boosted production of bioactive compounds under RB light may also be employed for delaying senescence and improving the shelf-life of fruits.

**Abstract:**

Phlegrean mandarin fruits are already known for health-promoting properties due to the high concentration of phytochemicals in peel, pulp, and seed. Biotic and abiotic factors, including light, may modulate their biosynthesis, metabolism, and accumulation. In this context, light-emitting diodes (LED) have recently been applied to control nutritional traits, ripening process, senescence, fruit shelf-life, and pathogenic microbial spoilage of fruits. This study investigated the effect of the seven-day exposure of Phlegrean mandarin fruits to two LED regimes, white (W) and red–blue (RB), to test the possibility that the storage under specific light wavelengths may be used as green preservation technology that enhances fruit phytochemical properties. To pursue this aim, the antioxidant activity and polyphenolic profile of the pulp and peel of mandarins under W and RB light regimes were evaluated and compared with Control fruits not exposed to LED treatment. Our results indicated that storage under W and RB treatments modulates the antioxidant content in pulp and peel differently. Compared to W, the RB regime increases the ascorbic acid, flavonoid, anthocyanin, and carotenoid concentrations, while the polyphenol profile analysis reveals that the number of important phytochemicals, i.e., quercetin rutinoside, chlorogenic acid, sinensetin, and rutin, are higher under W. The overall data demonstrated that postharvest LED irradiation is a valid tool for modifying fruit phytochemical properties, which also boosts specific bioactive compounds.

## 1. Introduction

Citrus crops are among the most cultivated crops in the world. The total production of citrus crops surpasses 100 million metric tons annually [1] for its health-promoting properties [2]. The Phlegrean mandarin (*Citrus reticulata* Blanco) is specifically cultivated in the Phlegraean volcanic zone of Naples (Italy). The unique properties of Phlegrean soil and climate make this cultivar an excellent source of phytochemicals, such as polyphenols, vitamin C, and flavonoids, with most of them being actively used in pharmaceutical applications as potential anti-COVID-19 molecules [3,4]. The concentration of bioactive compounds in mandarins strongly influences the preference of consumers who look for health-promoting foods. Furthermore, fruits rich in phytochemicals may represent the raw material to produce functional foods, nutraceuticals, or natural additives (such as antioxidant or antimicrobial agents) [5].

It is well known that the major loss of fruit nutritional properties and phytochemical content occurs during postharvest storage due to the rapid senescence and deterioration of tissues induced by the increase of reactive oxygen species (ROS) production. ROS are the main cause of cellular oxidative stress. Therefore, counteracting ROS production through antioxidants represents an effective way to delay the senescence of fresh products during storage [6]. The traditional postharvest techniques used for preserving fruits and vegetables are based on cooling or the application of chemical additives. Alternative and novel methods involve the use of Light Emitting Diodes (LEDs) in the postharvest. It is well documented that LED technology may enhance plant growth and photosynthesis, regulate senescence, improve plant and fruit shelf-life, and modulate the bioactive compound synthesis by setting suitable light quality regimes during plant development [7,8,9,10,11]. However, it must also be considered that the light quality effects on plant secondary metabolites are species-specific; therefore, the benefits obtained for one species may not occur for another species under the same light regime. For example, blue light on pepper strongly increased the flavonoid content in the epidermis [12]; in broccoli, red light treatment preserved the vitamin C during postharvest, while blue LED treatment increased carotenoid content [13]; in lettuce, supplemental red light regime during the growing season enhanced phenols concentration compared to white light [14].

The application of blue (470 nm) and red (660 nm) LED light at 20 °C for 6 days at a fluence rate of 50 W m^−2^ significantly increased some antioxidants in satsuma mandarin (*Citrus unshiu* Marc.) fruits, such as ẞ-cryptoxanthin [15].

Our previous research has revealed that Phlegrean mandarin is particularly rich in bioactive molecules compared to other commercial cultivars [16]. Furthermore, the peel and seeds of this specific cultivar are particularly rich in antioxidants [16]. The present study investigated the effects of the storage of Phlegrean mandarin fruits under two different light quality treatments: broad-spectrum white (W) and RB (red–blue), to assess if LED utilization in postharvest potentiates the content of phytochemicals also improving the conservation of fruits intended for marketing and human consumption.

Regarding conservation, it is a priority to maintain the quality of fresh products after harvest and to make sure they are long-lasting. Traditional postharvest preservation methods rely on cooling and chemical techniques [17]. Besides the enrichment of fruit nutritive quality, the use of LED technology during postharvest represents an eco-friendly tool for improving food shelf-life, fruit ripening control, and reducing microbial and fungi contamination of fruits and vegetables [18,19,20].

## 2. Materials and Methods

### 2.1. Plant Material, Sample Preparation, Storage Conditions, and Light Treatment

**Figure 1:**
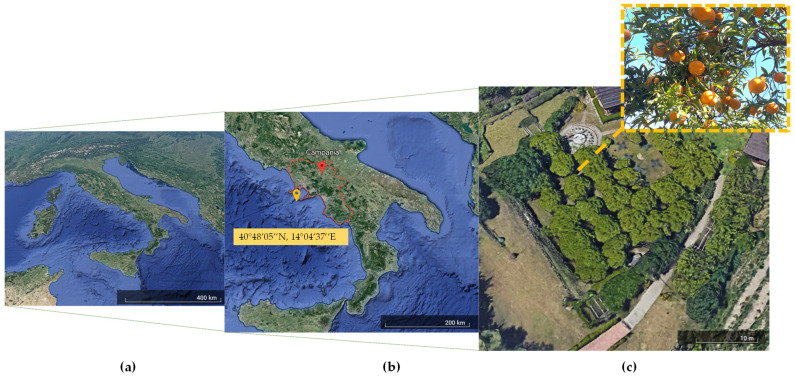
Map of the experimental site (40°48′05″ N, 14°04′37″ E) (Google Earth, earth.google.com/web/ (accessed on 1 June 2023)) located in the Phlegraean volcanic area of Naples, Regione Campania, Southern Italy (**a**,**b**), where *Citrus reticulata* Blanco is cultivated, and fruits were collected (**c**).

Fruits of *Citrus reticulata* Blanco (a traditional food product of Campania Region, Gazz. Uff. n° 43, 22 July 2015) are used in this study. This cultivar grows specifically in the Campania Region, Southern Italy. The experimental site is located at Bacoli, a city included in the Phlegraean volcanic zone of Naples (40°48′05″ N, 14°04′37″ E), as shown in Figure 1a–c, and is subjected to peculiar climate regime and soil characteristics [4].

Ripe mandarins were harvested in the field during the winter (early February) in the seasons 2021–2022. We randomly collected a total of three kilograms of fruits from different trees. The mandarins were placed in plastic bags and quickly transferred to the laboratory at the Department of Biology of the University of Naples Federico II for the following determinations.

**Figure 2:**
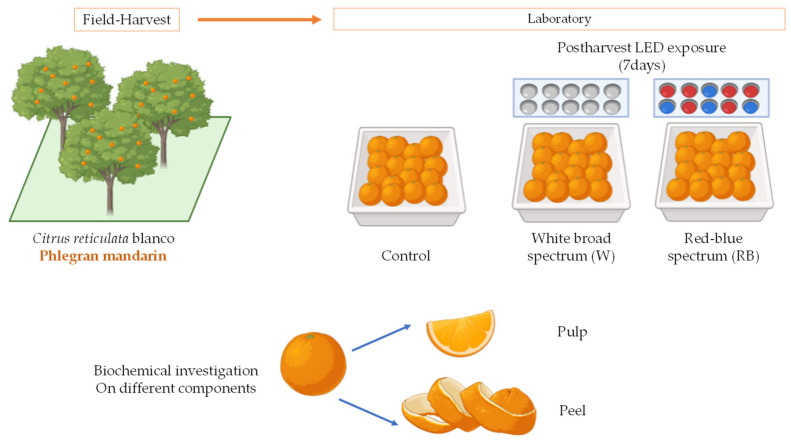
Experimental design. The fruits collected in the field were divided into three groups and exposed to W and RB light quality regimes provided by an LED source for 7 days. Not-exposed mandarins served as the Control. All biochemical investigations were performed on pulp and peel fruit components.

Then, the collected fruits were divided into three groups, each composed of eight fruits. The first group served as the Control and was not exposed to LED lighting system. The second and third groups were placed under two different light quality regimes: broad-spectrum white (W) and red–blue (R:B, 60:40, with emission peaks at 630 nm for red and 460 nm for blue), respectively (Figure 2).

**Figure 3:**
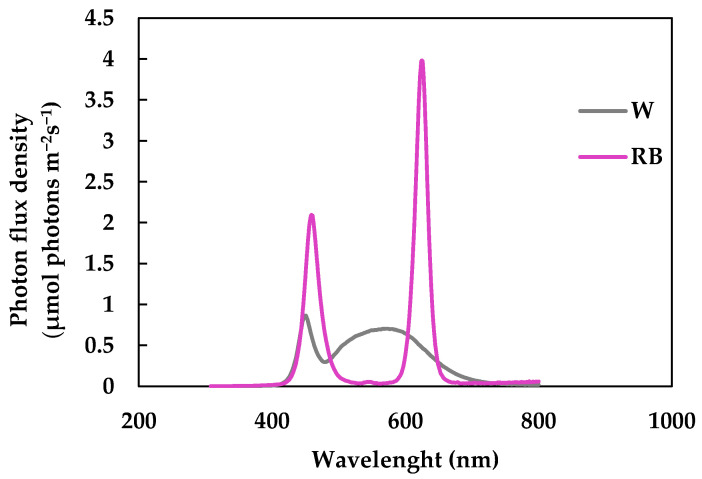
Light spectra set-up in the growth chamber for mandarin storage: broad-spectrum white (W) and red–blue light (RB). Emission peaks for red at 630 nm, emission peak for blue at 460 nm.

In detail, mandarins were positioned at 30 cm from the LED light source and continuously illuminated for one week using a photon flux density (PPFD) of 150 ± 20 μmol photons m^−2^ s^−1^. The PPFD and the spectral composition of the light regimes were determined by the SpectraPen mini radiometer (Photon System Instruments, spol. S.r.o., Czech Republic) (Figure 3).

Light treatments lasted seven days, and during light exposure, fruits were maintained at a temperature of 20 ± 2 °C. The selection and duration of the light regimes, as well as the temperature during light treatments, were based on previous studies performed on fruits and vegetables [11,17,21].

After treatments, fruits were cleaned with distilled water, divided into peel and pulp, and powdered with liquid nitrogen using a mortar and pestle. The fine powder was collected in glass test tubes and kept at −20 °C for the next analysis. A total of eight replicates were analyzed for both pulp and peel (a single fruit corresponds to one replica for a total of eight replicates). The analyses on fruits were carried out 7 days after the storage under LED treatments and on fruits not exposed to light quality regimes (Control).

### 2.2. Preparation of Methanolic Extracts

Peel and pulp were finely ground with liquid nitrogen using mortar and pestle. The extracts were prepared utilizing 0.20 g of pulverized sample in 2 mL of methanol 80% (*v*/*v*). The extracts were then centrifuged for 5 min at 11.000 rpm and stored at 4 °C for the following analyses. The extracts were utilized to quantify total polyphenols and flavonoids and measure the free radical scavenging activity by DPPH assay.

### 2.3. Total Polyphenol Concentration

Polyphenol concentration was measured according to Costanzo et al. [16]. An aliquot of sample diluted with methanol was mixed with Folin–Ciocalteu reagent. The solution was shaken, and an aliquot of 1.452 mL of sodium carbonate (Na_2_CO_3_) 700 mM was added. Samples were darkened for 2 h, and the absorbance was measured spectrophotometrically (UV-VIS Cary 100, Agilent Technologies, Palo Alto, CA, USA) at 765 nm. A gallic acid standard curve was used for the total polyphenol concentration calculation expressed as mg Gallic Acid Equivalents (GAE) g^−1^ FW.

### 2.4. Flavonoids Content Determination

Total flavonoid concentration was measured following Moulehi et al. [1] and Sun et al. [22]. Briefly, an aliquot (250 µL) of sample diluted in methanol was mixed with 75 µL of 5% sodium nitrite (NaNO_2_). Then, 150 µL of 10% aluminum chloride (AlCl_3_) and 500 µL NaOH 1 M and distilled water were added to the solution and up to the final volume of 1.525 mL. The absorbance of samples was measured at 510 nm. Total flavonoid concentration was calculated by means of a standard catechin curve and expressed as mg catechin equivalents per gram of fresh weight (mg CE g^−1^ FW).

### 2.5. Anthocyanin Determination

The anthocyanin content was assessed as reported in Mancinelli et al. [23] and Chung et al. [24]. Briefly, for anthocyanin extraction, 0.05 g of each sample was treated with 1% acidified methanol and kept 24 h in the dark at 4 °C. After centrifugation, supernatants were read spectrophotometrically at the wavelengths of 530 and 657 nm. The absorbance values were converted in concentrations using the extinction coefficient of 31.6 M^−1^ cm^−1^ in the following equation:

Anthocyanin content (µmol g^−1^) = [(A_530_ − 0.33 × A_657_)/31.6] × [volume (mL)/weight (g)].

### 2.6. Carotenoid and Ascorbic Acid Content Analysis

Total carotenoids in peel and pulp were estimated according to Lichtenthaler [25]. Afterwards, 100% ice-cold acetone was added to 0.010 g of powdered sample to allow the extraction of carotenoids. The extracts were then centrifugated (Labofuge GL, Heraeus Sepatech, Hanau, Germany) for 5 min at 5000 rpm. A spectrophotometer (Cary 100 UV-VIS, Agilent Technologies, Santa Clara, CA, USA) was used for determining the absorbance at the wavelength of 470 nm. Carotenoid concentration was expressed as mg g^−1^ fresh weight (FW).

The ascorbic acid (AsA) content was determined following the procedure reported by Costanzo et al. [4] using the Ascorbic Acid Assay Kit (MAK074, Sigma-Aldrich, St. Louis, MO, USA). Then, 0.010 g of powdered samples were treated with cold Ascorbic Acid Assay Buffer and centrifuged at 13.000 rpm for 10 min at 4 °C. The supernatant was mixed with the assay reagents provided by the kit. The AsA concentration was estimated by a colorimetric reaction. The absorbance was read at 570 nm using a microplate reader spectrophotometer (BioTek Synergy HTX, Software: Gen5^TM^ 2.07). The concentration of ascorbic acid was determined by a standard curve and expressed in ng μL^−1^.

### 2.7. Sample Antioxidant Activity Determination

#### 2.7.1. DPPH Method

Peel and pulp of mandarin extracts were also analyzed for free radical scavenging activity by the 1,1-diphenyl-2-picrylhydrazyl (DPPH) method described in Dudonné et al. [26]. A total of 67 µL of sample diluted in methanol was mixed with 2 mL of 6 × 10^−5^ M DPPH methanolic solution. The mixture was shaken and incubated at 37 °C for 20 min. The absorbance was read at a wavelength of 515 nm and converted in percentage of inhibition of DPPH radicals through the formula:

Inhibition (%) = [(Ab_blank_ − Ab_sample_)/Ab_blank_] × 100,

where Ab_blank_ is the blank absorbance; Ab_sample_ is the sample absorbance. Trolox was used as the reference Control.

#### 2.7.2. FRAP Method

The Ferric Reducing Antioxidant Power (FRAP) assay was performed as reported in George et al. [27]. Fine, powdered samples (0.25 g) were extracted in 5 mL of methanol/water solution (60:40 *v*/*v*) and then centrifuged at 14.000 rpm for 15 min at 4 °C after 1 h on ice. Successively, 150 µL of extract was added to 2.5 mL of 300 mM acetate buffer, 250 µL of 10 mM tripyridyltriazine (TPTZ), and 250 µL of FeCl_3_ (FRAP reagents) and incubated in the dark for 1 h. Sample absorbance was read spectrophotometrically at 593 nm (UV-VIS Cary 100, Agilent Technologies, Palo Alto, CA, USA). A Trolox standard curve was used for evaluating the antioxidant capacity expressed as µmol Trolox equivalents (µmol TE g^−1^ FW).

### 2.8. Liquid Chromatography with Tandem Mass Spectrometry (LC-MS/MS)

The LC-MS/MS was carried out following the procedure reported by Costanzo et al. [4]. Briefly, standard (stock) solutions were prepared by adding 1 mL aliquots of each analyte to a 10 mL volumetric flask, adjusting the volume with methanol to obtain 1000 µg L^−1^ of each analyte, and storing at −20 °C until the analysis.

Quantitative analysis was carried out by constructing calibration curves for a set of standard molecules selected for the different classes of analytes to be investigated. Standard mixtures were prepared through sequence dilution: 2.5-5-25-50-100-200 µg L^−1^.

Sample preparation: 5 g of fresh peel and 15 g of fresh pulp were weighed and ground, and for each, a solution of methanol, water, and acetonitrile (70:20:10, *v*/*v*/*v*) was added. Samples were then placed on a shaker in dark condition for 6 h. After that, the supernatant was centrifuged, filtered using 0.2 µm PFTE syringe filters, and diluted 1:10 (*v*/*v*) in the mobile phase. The supernatant was then directly transferred into an HPLC autosampler, and 1 µL of supernatant was analyzed in an LC-MS/MS assay.

The instrumentation and conditions of LC–MS/MS were the following: 1 μL of supernatant was analyzed by means of an AB-sciex 5500 QTRAP^®^ system with an HPLC chromatography system Exion LC™. Mixing eluent A (0.1% Formic Acid in water) and eluent B (0.1% Formic Acid in acetonitrile) was utilized to generate the mobile phase. The flow rate was set at 0.200 mL min^−1^. The chromatographic gradient was set from 20% B to 90% B after 4 min, detained for 2 min, and returned to 20% B after 1 min. Tandem mass spectrometry was achieved using a Turbo V^TM^ ion source operated in positive ion mode, and for the selected analytes, a multiple reaction monitoring (MRM) mode was utilized.

### 2.9. Statistical Treatment of Data

All data were analyzed and processed by software Sigma Plot 12.0 (Systat Software Inc., San Francisco, CA, USA). One-way ANOVA was used to evaluate the significant differences among treatments considering *p* < 0.05 as significance level. Duncan’s coefficient was performed to assess the multiple comparison tests. The Shapiro–Wilk test was used to check the normal distribution of data. All measurements are means ± standard error (SE) (*n* = 8).

## 3. Results

The analyses were carried out on 7-day harvested samples not exposed to LED (Control condition, C) and exposed to W and RB light regimes.

### 3.1. Total Phenolic Compounds in Control, W, and RB Fruits

#### Total Polyphenols, Flavonoid, and Anthocyanin Content

**Figure 4:**
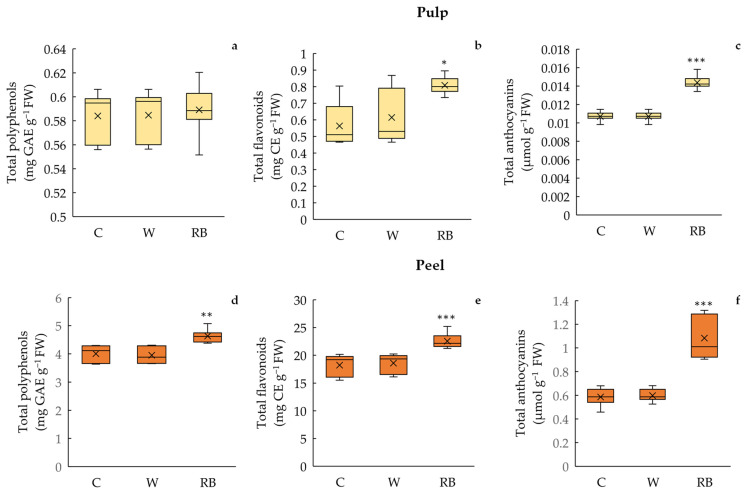
Total polyphenols (**a**,**d**), flavonoids (**b**,**e**), and anthocyanins (**c**,**f**) in pulp and peel of Phlegrean mandarin fruits not exposed (C) and exposed for 7 days to white (W) and red–blue (RB) light quality regimes. Data (means ± standard error *n* = 8) were represented by boxplots, where the height of the box is the interquartile range containing 50% of the observations, ranging between the first and third quartiles, the line inside the box is the median, and the cross symbol is the mean value; the whiskers extending from the box indicate the variability outside the upper and lower quartiles. Significant differences among treatments are indicated by asterisks according to one-way ANOVA test (* *p* < 0.05, ** *p* < 0.01; *** *p* < 0.001).

The total polyphenol content was not significantly different among C, W, and RB conditions in the pulp. Instead, in the peel, the polyphenol content was significantly higher (*p* < 0.01) in RB (peel: 4.63 ± 0.08 mg GAE eq g^−1^ FW; pulp: 0.59 ± 0.01 mg GAE g^−1^ FW) than C (peel: 4.00 ± 0.10 mg GAE eq g^−1^ FW; pulp: 0.59 ± 0.01 mg GAE g^−1^ FW) and W fruits (peel: 3.95 ± 0.10 mg GAE eq g^−1^ FW; pulp: 0.58 ± 0.01 mg GAE g^−1^ FW). No outliers in either pulp or peel were detected (Figure 4a,d).

Total flavonoid content varied significantly in peel (*p* < 0.001) and pulp (*p* < 0.05) in response to the different light quality regimes, showing the highest concentration in pulp and peel of fruits exposed to RB treatment (peel: 23.0 ± 0.48 mg Cat eq g^−1^ FW; pulp: 0.80 ± 0.02 mg Cat eq g^−1^ FW) compared to W (peel: 18.0 ± 0.61 mg Cat eq g^−1^ FW; pulp: 0.60 ± 0,05 mg Cat eq g^−1^ FW) and C (peel: 18.2 ± 0.68 mg Cat eq g^−1^ FW; pulp: 0.56 ± 0.04 mg Cat eq g^−1^ FW). No outliers were found for pulp and peel (Figure 4b,e).

Similarly, the anthocyanin concentration was higher (*p* < 0.001) in pulp and peel of RB mandarins (peel: 1.08 ± 0.06 µmol g^−1^ FW; pulp: 0.014 ± 0.000 µmol g^−1^ FW) compared to C (peel: 0.58 ± 0.02 µmol g^−1^ FW; pulp: 0.01 ± 0.00 µmol g^−1^ FW) and W (peel: 0.60 ± 0.01 µmol g^−1^ FW; pulp: 0.014 ± 0.000 µmol g^−1^ FW). No outliers were detected among sample groups (Figure 4c,f).

### 3.2. Total Carotenoids and Ascorbic Acid Content

**Figure 5:**
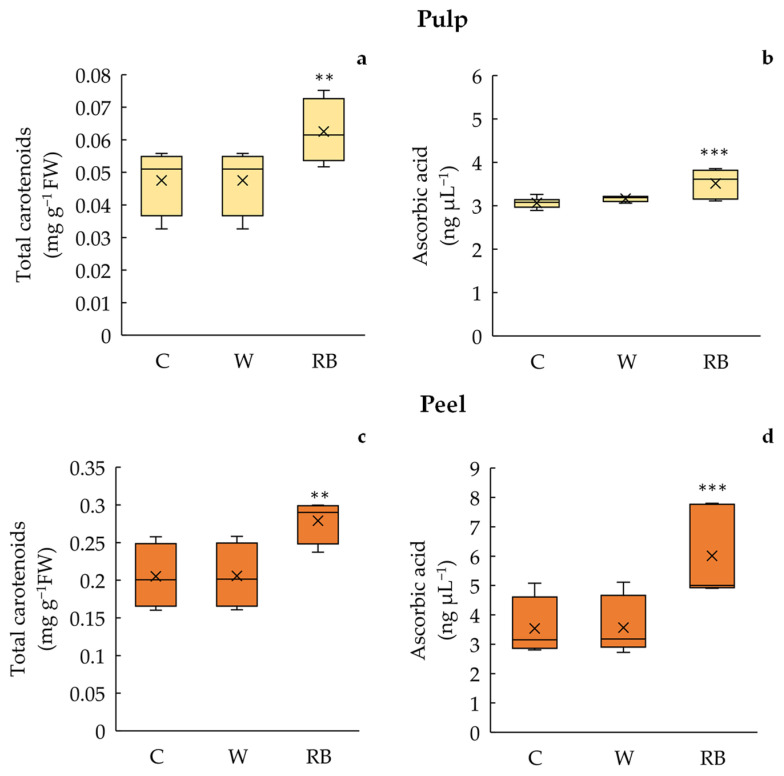
Total carotenoids (**a**,**c**) and ascorbic acid (**b**,**d**) content in pulp and peel of Phlegrean mandarin fruit not exposed (C) and exposed for 7 days to white (W) and red–blue (RB) light quality regimes. Data (means ± standard error *n* = 8) were represented by boxplots, where the height of the box is the interquartile range containing 50% of the observations, ranging between the first and third quartiles, the line inside the box is the median, and the cross symbol is the mean value; the whiskers extending from the box indicate the variability outside the upper and lower quartiles. Significant differences among treatments are indicated by asterisk according to one-way ANOVA test (** *p* < 0.01; *** *p* < 0.001).

Carotenoid content was significantly (*p* < 0.01) influenced by fruit storage under different light quality regimes both in peel and pulp (Figure 5a,c).

In particular, the values measured in peel and pulp for RB (peel: 0.28 ± 0.01 mg g^−1^ FW; pulp: 0.062 ± 0.003 mg g^−1^ FW) were higher than those found in C (peel: 0.20 ± 0.01 mg g^−1^ FW; pulp: 0.051 ± 0.003 mg g^−1^ FW) and W fruits (0.22 ± 0.01 mg g^−1^ FW; pulp: 0.047 ± 0.003 mg g^−1^ FW), without any outliers.

Similarly, the AsA concentration was higher (*p* < 0.001) in pulp and peel of RB fruits (peel: 6.01 ± 0.51; pulp: 3.51 ± 0.11) compared to C (peel: 3.54 ± 0.34; pulp: 3.07 ± 0.04) and W (peel: 3.57 ± 0.03; pulp: 3.16 ± 0.02) (Figure 5b,d).

### 3.3. Antioxidant Capacity and DPPH Radical Scavenging Activity

**Figure 6:**
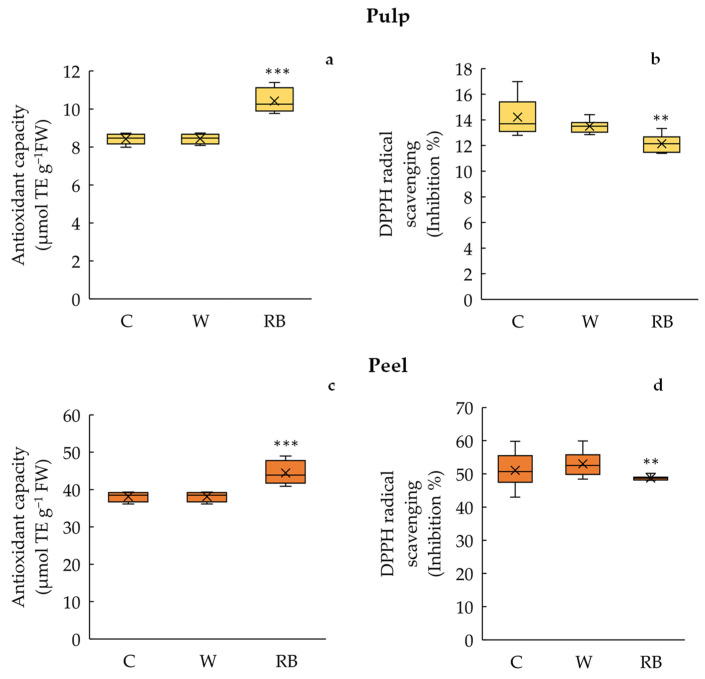
Antioxidant capacity (**a**,**c**) and DPPH radical scavenging activity (**b**,**d**) in pulp and peel of Phlegrean mandarin fruit not exposed (C) and exposed for 7 days to white (W) and red–blue (RB) light quality regimes. Data (means ± standard error *n* = 8) were represented by boxplots, where the height of the box is the interquartile range containing 50% of the observations, ranging between the first and third quartiles, the line inside the box is the median, and the cross symbol is the mean value; the whiskers extending from the box indicate the variability outside the upper and lower quartiles. Significant differences among treatments are indicated by asterisk according to one-way ANOVA test (** *p* < 0.01; *** *p* < 0.001).

The total antioxidant capacity in both pulp and peel was significantly higher (*p* < 0.001) in RB (peel: 44.4 ± 1.08 µmol TE g^−1^ FW; pulp: 10.4 ± 0.22 µmol TE g^−1^ FW) than in C (peel: 38.1 ± 0.45 µmol TE g^−1^ FW; pulp: 8.43 ± 0.09 µmol TE g^−1^ FW) and W fruits (peel: 38.1 ± 0.44 µmol TE g^−1^ FW; pulp: 8.42 ± 0.08 µmol TE g^−1^ FW). No outliers were evident in the three different groups (Figure 6a,c).

Conversely, the DPPH free radical scavenging activity was significantly higher (*p* < 0.01) in the pulp and peel of C (peel: 51.0 ± 1.88%; pulp: 14.0 ± 0.51%) and W (peel: 53.0 ± 1.33%; pulp: 13.0 ± 0.18%) than in RB fruits (peel: 48.7 ± 0.24%; pulp: 12.1 ± 0.25%) (Figure 6b,d).

### 3.4. Polyphenolic Profile of Pulp and Peel of W and RB Fruits

Since no statistically significant difference was found between Control and W fruits, the screening of polyphenolic profile was carried out by comparing mandarin pulp and peel only for W and RB conditions. Table 1 reported the quantitative polyphenol screening identified in both pulp and peel of fruits exposed to W and RB light treatments.

Concerning phenols exceeding the concentration of 0.1 µg g^−1^, the pulp of RB mandarins was richer in caffeic acid, haesperetin, ferulic acid (*p* < 0.01), and kaempferol 3-O-rutinoside (*p* < 0.05) than W mandarins and, in concomitance, less wealthy in caffeoylquinic acid derivative, quercetin rutinoside, chlorogenic acid, isorhoifolin, naringenin-7-O-neohesperidoside, delphinidin rutinoside, sinensetin (*p* < 0.01), valoneic acid dilactone, and rutin (*p* < 0.001).

Compared to mandarins treated under W light, those exposed to RB regime showed, in the peel, higher concentrations of vanillic acid, caffeic acid, pelargonidin, methyl gallate, EfisetinidolEC isomer 3, ferulic acid, linarin, puerarin, gallic acid (*p* < 0.001), delphinidin, EGCepicatechin dimer, astragalin, coumaric acid, caffeine, cyanidin-3-O-glucoside, delphinidin rutinoside, and sinensetin (*p* < 0.01). Conversely, the peel of W mandarins was richer in quercetin, kaempferol3Orutinoside, caffeoylquinic acid derivate, naringin, haesperetin, isorhoifolin, hyperoside, naringenin7Oneohesperidoside (*p* < 0.001), chlorogenic acid, 3PCoumaroylquinic acid, quercitin-3-glucoside, quercetin hexoside, isorhamentin3ONeohesperidoside, quercitin-3-glucoside, valoneic acid dilactone (*p* < 0.01), naringin, haesperetin, orientin, and peonidin-3-O-glucoside (*p* < 0.05).

## 4. Discussion

During postharvest storage, most fruits and vegetables deteriorate, losing several nutraceutical and organoleptic characteristics.

ROS (Reactive Oxygen species) represent the leading cause of plant senescence, influencing several physiological processes and cell structural components such as lipids and proteins. Therefore, counteracting free radicals’ production ensures safeguarding cellular functions and delaying the deterioration process of tissues and fruits [6].

Our study demonstrated that the storage of mandarin fruits to W and RB light regimes after harvesting produced significant changes in antioxidant compounds, confirming that LED light treatments were an effective tool to preserve fruits in the postharvest when the deteriorating process begins. In particular, our results showed that the RB light stimulates the synthesis of total polyphenols, carotenoids, ascorbic acid, and antioxidant activity in mandarin fruits during storage, allowing their preservation without the employment of chemicals and improving both shelf-life and nutritional quality.

Our data agree with other studies which demonstrate that different light quality regimes, monochromatic or in combination, were often utilized to maximize crop production, nutritional quality, food preservation, and pathogenic microbial prevention [13,28,29].

Among phytochemicals that are affected by light quality regimes, we focused the attention on some specific phenolic compounds involved in plant defense against biotic and abiotic stresses, which may also be assumed by humans with diet [30,31].

It has been reported that RB treatment stimulated the synthesis of polyphenols, anthocyanins, and flavonoids in onion [32] and strawberry fruits [7,8], while the exposure of citrus fruits (*Citrus* sp.) to blue light during storage favored the phenolic compound accumulation [33].

Consistent with these studies, our results showed a significant increase of flavonoids (~32%) and anthocyanins (~81%) in the pulp of mandarins stored under the RB compared to W and Control fruits. A recent paper on the Phlegrean mandarin cultivar has demonstrated that in fresh fruits, phenolic compound concentration did not change in the pulp, while it decreased in the peel with ripening [4]; our study indicated that if ripe fruits are preserved for seven days under RB light not only the polyphenol level but also flavonoids and anthocyanin amount significantly increase in both pulp and peel compared to Control and W light treatment, validating the effectiveness of this method for citrus fruit storage.

We suppose that the combination of red and blue wavelengths (R:B, 60:40) used in RB treatment and the ripening may have stimulated the activation of biosynthetic pathways for the production of phenolic substances in fruit. It has been demonstrated that light intensity and fruit maturity strongly impact antioxidant capacity and phytonutrient levels in red raspberries [34]. Moreover, the phenylalanine ammonia-lyase enzyme (PAL), involved in the first phase of the phenylpropanoid pathway [35], as well as the chalcone synthase flavanone-3-hydroxylase and anthocyanidin synthase enzymes involved in the synthesis and accumulation of polyphenols, flavonoids, and anthocyanins [9] are susceptible to light quality.

The qualitative analysis of the phenolic fruit profile provides detailed information on phenolic compounds, especially flavonoids, up- or down-regulated by light quality treatments. In the peel and pulp of RB fruits, some flavones, flavanols, anthocyanidins, and benzoic and cinnamic acids increased, suggesting that these specific molecules mainly contributed to phenolic accumulation under RB treatment [36], which likely has acted on the phenylpropanoid pathways involved in the biosynthesis of some flavonoids, i.e., sinensetin, and linarin, especially in the peel. The augment of such compounds is worthy of attention because sinensetin has been recognized to have potent antioxidant, anti-inflammatory, anticancer, and antimicrobial activity [37]. At the same time, linarin exhibited a potential action against central nervous system disorders, osteoarthritis, and osteoporosis [38].

Interestingly, the storage under RB also boosted cinnamic acids and anthocyanidins. Cinnamic acids such as caffeic, ferulic, and vanillic are known for a wide range of pharmacological benefits for human health due to anti-inflammatory, antimicrobial, cardioprotective, anticancer, and antidiabetic properties [39,40]. The more abundant anthocyanidins were delphinidin, pelargonidin, delphinidin-3-O-glucoside, cyanidin-3-O-glucoside, and delphinidin rutinoside—compounds particularly active in preventing cardiovascular and neurodegenerative diseases [41]. The over-production of anthocyanidin under RB treatment likely may depend on the blue light-mediated activation (through cryptochromes) of specific enzymes such as chalcone synthase (CHS) and dihydroflavonol-4-reductase (DFR) involved in the biosynthetic pathways of flavonoid/phenolics metabolites [42].

Interestingly, fruit storage under W light has stimulated the production of other flavonoids, such as rutin and quercetin. Several studies demonstrated that blue wavelengths prevent the synthesis of these molecules [43] by inhibiting the expression of miR393, miR394, and miR395 genes of their synthetic pathway [44]. In our case, it is likely to suppose that the W regime, having a reduced amount of blue wavelength (21%, data not showed) compared to RB treatment (40%), did not induce alteration in rutin and quercetin synthetic route, conferring other kinds of benefits to W fruits.

Indeed, rutin has neuroprotective properties for treating neurodegenerative diseases, whereas quercetin is a promising molecule against COVID-19. The pulp and peel of W mandarins are also richer in other flavonoids such as naringin, mainly involved in bone regeneration and treatment of metabolic syndrome and central nervous system diseases [45]; hesperidin with hypolipidemic, antidiabetic, and cardioprotective action; valoneic acid with antidiabetic activity [46]; and chlorogenic acid with anti-obesity, hepatoprotective, and anti-hypertension properties [47].

We have supposed that abundance of some polyphenols in W, absent or negligible in RB fruits, may also depend on the presence of green wavelengths in the white spectrum. According to this hypothesis, some authors have found higher concentrations of rutin and chlorogenic and caffeic acid in blueberry fruits after three days of storage under red–green–blue light treatment [48].

Specific light quality regimes also modulate the expression of light receptors involved in carotenoid biosynthesis pathways, affecting the nutritional value of fruits and vegetables [9,14]. The enzyme which limits the rate in the biosynthetic pathway of carotenoids is the phytoene synthase (PSY); the PSY encoding gene is regulated by light [43]. The expression of the PSY is generally stimulated by blue light leading to the production of beta carotene in *Citrus unshiu* marc [15]. On the other hand, red wavelengths enhanced the activity of lycopene-β-cyclase, β-carotene hydroxylase, and violaxanthin de-epoxidase enzymes [49]. Generally, the RB treatment stimulates fruit carotenoid production (beta carotene and lycopene) more than monochromatic wavelengths [43,50,51]. Citrus fruits (*Citrus unshiu* Marc) exposed to red and blue LED light for 6 days showed a significant increase of ẞ-cryptoxanthin [15]. Consistent with these findings, the storage of Phlegran mandarin under RB light increased the carotenoid content in pulp (~36%) and peel (~32%) compared to W and Control, amplifying the already known recognized properties of this citrus cultivar compared to the others [4,16].

Fresh mandarins stored under RB light also showed a higher ascorbic acid concentration than W and Control fruits confirming the efficiency of blue light, alone or combined with red wavelengths, in stimulating the expression of genes related to AsA metabolism as found in other species, like soybean, broccoli, and lettuce [10,13,52].

Therefore, the higher amount of carotenoids and ascorbic acid encourages the consumption of fresh mandarin fruits stored under RB light without risks associated with the loss of nutraceutical benefits for consumers.

According to other studies [16,53,54], it is a likely assessment that our results indicated that phenolic compounds contribute significantly to antioxidant activity. Total antioxidant capacity (FRAP) was considerably higher in fruits under RB light in both peel and pulp (~16% and 23%, respectively) compared to Control and W treatments. The opposite trend was observed for free radical scavenging activity (DPPH%), higher in the pulp and peel of Control and W mandarins (~11% and ~9%, respectively). As Hasan et al. [55] reported, the increased antioxidant activity could result from continuous exposure of fruits to LEDs, regardless of the wavelengths. Moreover, the opposite behavior found between FRAP and DPPH assays perfectly matched the results of qualitative analysis, which showed that some phenolic compounds increased in fruits under RB, while others increased in response to exposure to W light treatment. It must be considered that the antioxidant activity of phenolic compounds depends on the proton-donating ability related to their structure. Therefore, the efficiency of these molecules as free radical scavengers can vary according to the number of hydroxyl groups, their position on the molecule, the presence of glycosylation, and the proximity of carboxylate and hydroxyl groups on the phenolic ring.

Besides the benefits of nutraceutical properties, the induction of phenol profile modification using LEDs in postharvest storage represents a valuable approach to delay senescence and prevent fruit deterioration [9,33].

The illumination with blue or red LED light inhibited water loss in navel orange fruit, representing the main cause of the decrease in fruit firmness. Peel composition, including the number of scavenger molecules, has a key role in controlling fruit water loss [29] and maintaining fruit freshness [56]. Based on this evidence, using RB-LED treatment during Phlegrean mandarin storage, the boosted antioxidants produced in the peel may help to preserve fruits from deterioration. A good peel conservation status can be helpful to guarantee the overall fruit freshness, shelf-life, and nutritional properties. Furthermore, considering the specific properties of the peel of Phlegrean mandarin compared to other cultivars, the maintenance of essential constituents, in terms of phytochemicals, can elevate this specific fruit part from waste into a valuable bioproduct, exploitable in pharmaceutical, cosmetical, and agronomic fields [4,57].

## 5. Conclusions

In this study, the increase of beneficial metabolites, i.e., phenolic compounds, carotenoids, ascorbic acid, and antioxidant activity in citrus fruits exposed to RB light, confirmed the effectiveness of LED utilization in fruit storage. Our results evidenced how specific light quality treatments may be used as an efficient strategy for extending conservation and nutritional value in mandarin fruits. Additionally, the analysis of the polyphenolic profile highlighted how the storage under W or RB improves the concentration of linarin, sinensetin, vanoleic acid, naringin and derivates, and cinnamic acids and derivates in the peel and pulp of Phleagran mandarin without the occurrence of biotechnological or chemical manipulations.

The overall results demonstrated that postharvest LED irradiation might be a suitable green preservation technology to strengthen the phytochemical properties in the specific Phlegrean mandarin cultivar, which is already appreciated for its unique antioxidant properties.

## Figures and Tables

**Table 1:** Polyphenolic profile of pulp and peel of Phlegrean mandarin exposed to W and RB light quality regimes for 7 days from harvest. Data are expressed in µg g^−1^ FW as means ± standard error (SE) (*n* = 3). Significant differences among treatments are indicated by asterisk according to *t*-test (ns, not significant; * *p* < 0.05; ** *p* < 0.01; *** *p* < 0.001).

	Pulp		Peel	
Compound	W	RB	W	RB
Naringin	156.798 ^ns^	143.395 ^ns^	187.742 *	63.560
Quercetin	<0.1	<0.1	0.740 ***	0.173
Vanillic acid	2.465 ^ns^	2.884 ^ns^	16.320	29.024 ***
Caffeic acid	4.442	4.766 **	15.166	22.893 ***
Delphinidin	<0.1	<0.1	0.106	0.903 **
Pelargonidin	<0.1	<0.1	<0.1	1.657 ***
Haesperetin	0.166	0.360 **	2.714 *	2.150
Kaempferol 3-O-rutinoside	29.931	33.867 *	127.695 ***	95.175
Caffeoylquinic acid derivatives	1.802 **	0.907	231.196***	35.454
Quercetin rutinoside	0.252 **	0.202	35.031 ^ns^	34.395 ^ns^
Methyl gallate	<0.1	<0.1	0.279	0.533 ***
EGC epicatechin dimer	<0.1	<0.1	1.420	2.649 **
EfisetinidolEC isomer 3	<0.1	<0.1	<0.1	2.072 ***
Chlorogenic acid	35.144 **	15.001	2311.387 **	2148.683
Ferulic acid	0.140	0.318 **	0.857	3.399 ***
Linarin	<0.1	<0.1	<0.1	0.130 ***
Puerarin	<0.1	<0.1	<0.1	0.192***
Diosmetin	<0.1	<0.1	0.105 ^ns^	0.108 ^ns^
Astragalin	<0.1	<0.1	0.246	1.354 **
Coumaric acid	1.719 ^ns^	1.400 ^ns^	3.869	9.582 **
3-p-coumaroylquinic acid	<0.1	<0.1	3.050 **	1.736
Orientin	<0.1	<0.1	5.427 *	3.770
Isorhoifolin	0.503 **	0.315	9.447 ***	1.870
Hyperoside	<0.1	<0.1	0.771 ***	<0.1
Quercetin hexoside	<0.1	<0.1	1.070 **	0.710
Nicotinflorin	22.905 ^ns^	20.713 ^ns^	133.682 ^ns^	121.503 ^ns^
Naringenin-7-O-neohesperidoside	79.538 **	41.186	150.450 ***	27.212
Isorhamnetin 3-neohesperidoside	2.765 ^ns^	2.470 ^ns^	8.529 **	5.540
Caffeine	<0.1	<0.1	1.497	1.861 **
Gallic acid	<0.1	<0.1	<0.1	0.269 ***
Delphinidin-3-O-glucoside	<0.1	<0.1	<0.001	0.669 **
Cyanidin-3-O-glucoside	<0.1	<0.1	0.066	0.104 **
Peonidin-3-O-glucoside	<0.1	<0.1	2.300 *	< 0.1
Delphinidin rutinoside	0.138 **	<0.1	0.842	1.524 **
Quercetin-3-glucoside	<0.1	<0.1	1.894 **	0.2506
Quercetin 3-O-rhamnoside	<0.1	<0.1	<0.1 ^ns^	<0.1 ^ns^
Valoneic acid dilactone	0.519 ***	<0.1	7.839 **	0.171
Sinensetin	0.108 **	<0.1	37.807	58.344 **
Rutin	0.141 ***	<0.1	1.613 ^ns^	1.639 ^ns^

## Data Availability

Data are available from the corresponding authors upon reasonable request.
